# Relation of STAT3 rs1053005 Variation and miR-452-3p with Osteoarthritis Susceptibility and Severity and the Clinical Response to High-Molecular-Weight Hyaluronic Acid Injection in Osteoarthritis Patients

**DOI:** 10.3390/diagnostics13233544

**Published:** 2023-11-27

**Authors:** Alaa S. Wahba, Dina A. Mohamed, Mohamed T. Mehanna, Noha M. Mesbah, Dina M. Abo-elmatty, Eman T. Mehanna

**Affiliations:** 1 Department of Biochemistry, Faculty of Pharmacy, Suez Canal University, Ismailia 41522, Egypt; alaa.samir@pharm.suez.edu.eg (A.S.W.); dina_hakim@pharm.suez.edu.eg (D.A.M.); noha_mesbah@pharm.suez.edu.eg (N.M.M.); dina_abouelmouti@pharm.suez.edu.eg (D.M.A.-e.); 2 Department of Orthopedic Surgery and Trauma, Faculty of Medicine, Suez Canal University, Ismailia 41522, Egypt; m.taha.mehanna@med.suez.edu.eg

**Keywords:** osteoarthritis, STAT3, miR-452-3p, rs1053005, HMW-HA injections

## Abstract

Polymorphisms in the 3′ untranslated region of STAT3 mRNA can derange STAT3 gene expression via modifying the microRNA-binding site. This study aimed to examine the impact of STAT3 rs1053005 variation and miR-452-3p expression on osteoarthritis (OA) susceptibility and severity and the efficacy of intra-articular high-molecular-weight hyaluronic acid (HMW-HA) injection as a therapy option for knee OA. Two hundred and fifty-eight OA patients and 200 healthy controls were enrolled in the study. STAT3 genotyping and STAT3 and miR-452-3p expression were carried out using allelic-discrimination PCR and quantitative real-time PCR. Functional assessment and pain evaluation were performed for all patients. Eighty-three patients received HMW-HA injections, and multiple follow-up visits were performed. STAT3 mRNA was upregulated, and expression was positively associated with plasmin, TNF-α, MMP-3, and STAT3 serum levels, whereas miR-452-3p was downregulated and negatively associated with the previously mentioned parameters in OA patients. Osteoarthritis patients had a lower prevalence of the minor allele of the rs1053005 variant (*p* < 0.001). Plasmin, TNF, MMP-3, and STAT3 mRNA and protein levels were significantly decreased, and miR-452-3p expression was significantly increased in the GG genotype compared to AG and AA genotypes. HMW-HA injection improved OA patients’ clinical scores with concomitant decreased STAT3 levels and enhanced expression of miR-452-3p. More efficient improvement was observed in rs1053005 AG + GG genotype carriers vs. AA genotype carriers. The G allele of STAT3 rs1053005 (A/G) polymorphism was associated with decreased OA susceptibility and severity and enhanced clinical response to HMW-HA injection, possibly via enhancing miR-452-3p binding and a subsequent decrease in STAT3 expression.

## 1. Introduction

Osteoarthritis (OA) is a chronic degenerative joint disease, characterized by progressive degradation and loss of articular cartilage, subchondral bone sclerosis, inflammation, and osteophyte formation [1]. Limited joint movement, edema, and chronic pain are the major signs of OA. Recent studies have shown that it affects 240 million people globally and is considered a main factor contributing to pain, disability, and decreased adult work performance throughout the world [2]. There are two types of OA: primary and secondary. Primary OA is more prevalent and is a result of aging, whereas secondary OA is caused by joint trauma or other illness [3]. OA can be regarded as a multifactorial disease, with several factors including aging, genetic predisposition, epigenetic control, inflammation, obesity, and trauma being involved in its pathophysiology [4]. 

Pro-inflammatory cytokines such as interleukin (IL)-1β, IL-6, IL-15, IL-17, IL-18, and tumor necrosis factor-alpha (TNF-α) were found to be elevated in areas of synovial, cartilage, and subchondral bone of OA patients. These cytokines can trigger upregulated expression of catabolic enzymes such as plasmin and matrix metalloproteinases (MMPs) that break down the extracellular matrix [5,6,7]. Signal transducer and activator of transcription 3 (STAT3) has been described as one of the critical factors involved in the initiation and development of inflammatory responses in OA. Previous studies have provided evidence that microRNAs (miRNAs) play a major role in the regulation of STAT3 expression through binding to the 3′ untranslated region (3′UTR) of STAT3 mRNA, with single-nucleotide polymorphisms (SNPs) in the miRNA-binding site influencing their regulatory activity [8,9]. miR-452-3p, found on human chromosome Xq28, is abnormally expressed in various disorders [10]. Downregulated miR-452 has been previously reported to be associated with STAT3 activation and increased inflammation [11]. Importantly, rs1053005 A/G SNP in the 3′UTR of STAT3 mRNA was previously reported as a susceptibility locus for various disorders [12,13,14,15]. This study aimed to elucidate the potential association of rs1053005 STAT3 SNP with susceptibility to OA, with a possible influence of miR-452-3p expression.

The polygenic nature of OA, together with the involvement of multiple molecular mechanisms, contribute to a lack of efficacy of current therapies to reverse or slow down the disease progression [16]. It is crucial to use a therapeutic approach that incorporates physical therapy, non-steroidal anti-inflammatory drugs, analgesics, rehabilitation, chondroprotective agents, and intra-articular treatment with infiltrative substances like steroids and hyaluronates as part of the overall management of patients with OA [17]. 

Hyaluronic acid (HA) is a non-sulfated glycosaminoglycan made up of units of D-glucuronic acid and N-acetylglucosamine that alternately repeat, and it is naturally found in various animals’ tissues. The extracellular matrix (ECM) of soft connective tissues contains the highest levels of HA in the human body. Intracellular adhesion molecule (ICAM-1), cluster determinant 44 (CD44), and the receptor for hyaluronate-mediated motility (RHAMM) are a few examples of HA-specific receptors [18]. 

Binding with such receptors triggers various intracellular signal events, such as cytokine release and the stimulation of cell cycle proteins, and cell functional activities, such as cell migration and proliferation [19]. The viscoelastic characteristics of synovial fluid can be restored by intra-articular therapy with exogenous HA, since osteoarthritic joints have a lower concentration of HA than healthy joints. Exogenous HA can improve the production of proteoglycans and endogenous HA with chondrocytes, which help to regenerate cartilage and stop it from degrading [20]. Additionally, it can decrease the synthesis of matrix metalloproteinases and proinflammatory mediators as well as the nerve impulses and sensitivity linked to OA pain [17]. Although intra-articular injection of HA or its derivative is currently approved by the Food and Drug Administration only for patients with knee OA, there is conflicting evidence of its efficacy [21]. Hermans et al. [22] concluded that adding intra-articular high-molecular-weight hyaluronic acid (HMW-HA) to usual care is effective for knee OA in patients of working age (18–65 years).

Herein, we investigated the potential influence of rs1053005 STAT3 SNP on STAT3 mRNA and protein levels and the susceptibility and severity of OA. A chief objective was to assess miR-452-3p expression and its correlation with STAT3 levels and OA clinical characteristics in OA patients and rs1053005 genotypes. Another aim was to evaluate the association of rs1053005 STAT3 SNP with the therapeutic response to HMW-HA intra-articular injection in OA patients.

## 2. Materials and Methods

### 2.1. Study Participants

The study included 458 participants (258 unrelated OA patients and 200 age- and gender-matched healthy controls). Patients with OA were recruited from outpatient orthopedic clinics, Suez Canal University Hospitals, Ismailia, Egypt. Using the Kellgren and Lawrence (KL) osteoarthritis grading system illustrated in Table 1, patients were diagnosed with osteoarthritis through history taking, clinical examinations, and radiological tests [23]. All patients were chosen within grade 1, 2, or 3. 

Functional assessment was performed for all patients on the first outpatient visit using the Knee injury and Osteoarthritis Outcome Score (KOOS). Scores were transformed to a 0–100 scale, with 0 representing extreme knee problems and 100 representing no knee problems [24]. Pain was evaluated by the Numeric Rating Scale (NRS), resulting in a score between 0 (no pain) and 10 (most severe pain) [25]. The mean values of the clinical scores of all patients (*n* = 258) are represented in Table 2.

The recruitment of patients and controls was performed during the period from March to October 2022. Gout, ankylosing spondylitis, septic arthritis, rheumatoid arthritis, and other types of arthritis, as well as any other autoimmune or systemic inflammatory diseases (such as systemic lupus erythematosus (SLE), systemic sclerosis, spondyloarthropathies, psychotic bipolar disorder, inflammatory bowel disease, polymyositis, and dermatomyositis) were excluded from the study. Controls with no history of OA or any other autoimmune, inflammatory, or chronic diseases and no family history of autoimmune diseases participated in the study. Because all the study’s participants belonged to the same ethnic group, population stratification was less likely. The sample size was determined using a power of 80% and a level of significance of *p* < 0.05 [26].

### 2.2. Treatment and Intervention

All patients received the usual treatments, including NSAIDS or acetaminophen as well as health education programs, lifestyle modifications, physiotherapy programs, and bracing. Patients were divided into 2 groups: the HMW-HA group (*n* = 83) received a single injection of Antalvisc© containing 60 mL HMW-HA and 15 mL Mannitol (Novatex Bioengineering SAS, Paris, France) after consent. The No HMW-HA group (*n* = 175) did not receive HMW-HA injections. 

Intra-articular HMW-HA injections were recommendable for patients of working age (between 18–65 years old), with pain >3 months, mean pain severity ≥2 on the NRS, and KL grade I to III. Exclusion criteria for HMW-HA injections in this study included intra-articular HA injections within one year, intra-articular steroid injection within 3 months, (planned) pregnancy or lactation, inflammatory or septic arthritis, non-knee-related regular analgesic use, daily oral steroid therapy, poor general health, and patients unable to attend follow-up. Follow-up outpatient visits for patients in both groups (HMW-HA and No HMW-HA) were scheduled at baseline (before injection) and periodically for 6 months. Clinical and biochemical follow-up with blood samples being tested for STAT3 and miR-452-3p levels was performed at the ultimate follow-up (six months after the injection).

### 2.3. Ethical Approval

This study was approved by the Suez Canal University Faculty of Pharmacy’s Ethics Committee (202110MH1) and was conducted in accordance with the Declaration of Helsinki’s (2000 revision) principles. A written informed consent form was signed by each participant.

### 2.4. Biochemical and Immunochemical Analysis

Five milliliters of venous blood were drawn from each participant and divided into two portions. Two milliliters were collected in potassium EDTA tubes and used to extract DNA. The remaining three milliliters were collected in a plain tube for serum separation. Serum was used for RNA extraction, with subsequent gene expression analysis and evaluation of plasmin, MMP-3, TNF-α, and STAT3. After a follow-up period of six months, a 3 mL blood sample was drawn from all patients for serum separation, which was also used for RNA extraction and evaluation of STAT3 protein levels. 

Serum levels of plasmin, MMP-3, and TNF-α were measured using human-specific ELISA kits (BioSource International, CA, Cat. No: MBS026652, Cat No. MBS454323 and Cat No. MBS2502004, respectively). STAT3 serum levels were measured using Bioassay Technology, China (Cat. No. E0650Hu)**.** ELISA kits were utilized in accordance with the manufacturer’s instructions.

### 2.5. STAT3 mRNA and miR-452-3p Expression Analysis

Using the Qiagen miRNeasy mini kit (50) (Qiagen, Hilden, Germany, Cat. No: 217004) and following the manufacturer’s directions, total RNA including small RNA was isolated from serum samples. A NanoDrop 1000 spectrophotometer (NanoDrop Tech, Wilmington, DE) was utilized for determination of extracted RNA concentration. Using the GoTaq^®^ 1-step RT-qPCR kit supplied by Promega, Madison, WI, USA and the StepOnePlus™ real-time PCR thermal cycling device (Applied Biosystems, San Francisco, CA, USA), quantitative real-time PCR was performed to determine gene expression of STAT3 and miR-452-3p. The expression levels of STAT3 and miR-452-3p were normalized to GAPDH and U6, respectively. The sequences of primers and their annealing temperatures are listed in Table 3.

The final reaction volume was 20 µL and consisted of the following: 4 µL of RNA template (approximately 100–200 ng), 1 µL of each of the forward and reverse primers (200 nM) for both assessed and reference genes, 0.4 µL of GoScriptTM RT mix for 1-step RT-qPCR, 10 µL of GoTaq^®^ qPCR master mix, 0.31 µL of supplemental CXR reference dye, and 3.29 µL of nuclease-free water. The PCR protocol consisted of 15 min of reverse transcription at 37 °C, 10 min at 95 °C to stop the reverse transcriptase, and 40 cycles of denaturation at 95 °C for 10 s, annealing for 30 s, and extension at 72 °C for 30 s. ΔΔCt and fold change were calculated for all samples. Ten percent of the samples were chosen at random and reassessed in separate runs to test the qPCR’s reproducibility.

### 2.6. Genotyping 

The Wizard genomic DNA purification kit (Promega, Madison, USA) was used for genomic DNA extraction from blood, as directed by the manufacturer. The extracted DNA concentration and purity were measured using a NanoDrop ND-1000 (NanoDrop Tech., Inc., Wilmington, DE, USA). Genotyping of the STAT3 rs1053005 polymorphism was performed by RT-PCR. A final reaction volume of 20 µL was used for the PCR and contained 20 ng genomic DNA diluted to 9.5 µL with DNase-RNase-free water, 10 µL TaqMan universal PCR master mix, No AmpErase UNG (2x), and 0.5 µL 20X TaqMan SNP genotyping assay mix (Applied Biosystems, CA, USA, assay ID C_7530672_10). Each run included no-template controls (no DNA) and internal positive controls. PCR amplification and allelic discrimination were performed using ABI Prism 7900HT and the sequence detection system (SDS) software version 2.1.1 (Applied Biosystems, CA, USA). The PCR settings were applied as follows: a single cycle of initial denaturation (the holding stage for polymerase activation) at 95 °C for 10 min, 45 cycles of denaturation at 95 °C for 15 s and annealing/extension at 60 °C for 1 min. To completely remove the possibility of incorrect genotype calls, 10% of randomly chosen samples underwent reassessment in separate runs.

**Figure 1:**
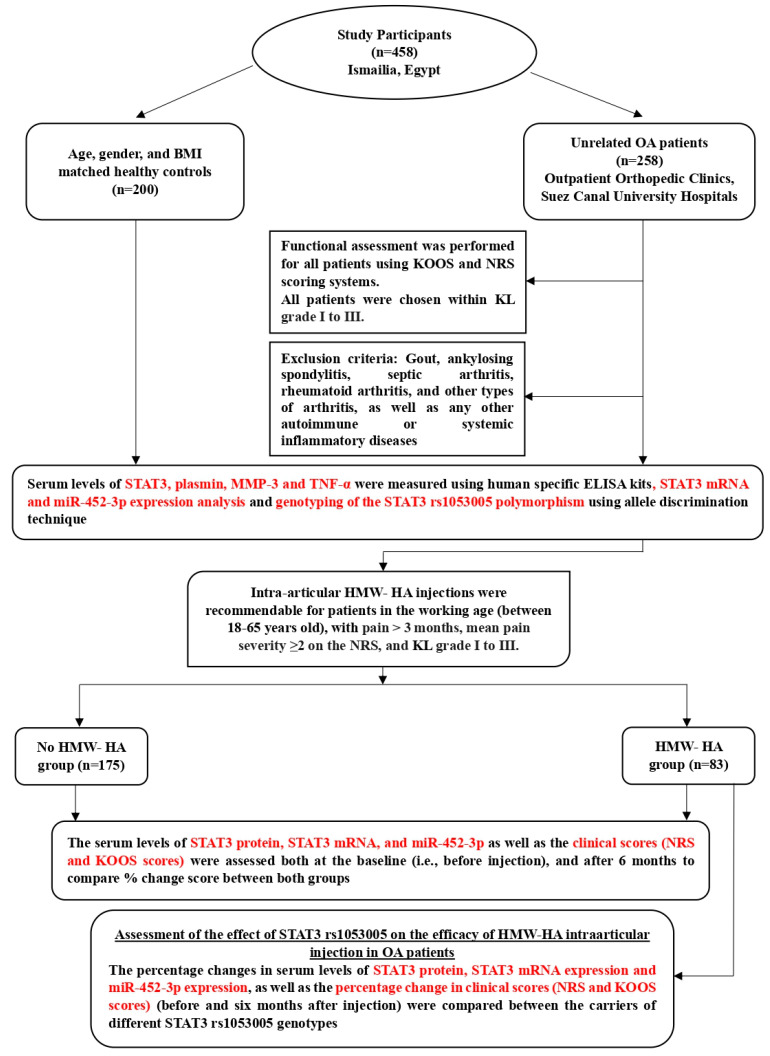
A flowchart representing the design of the study and the assessed parameters.

The study design and the applied measurements are summarized in Figure 1.

### 2.7. Statistical Analysis

Statistical Package for Social Sciences, version 25.0 software (SPSS Inc., Chicago, IL, USA) was used to conduct the statistical analysis. For quantitative parametric variables, descriptive statistics were displayed as mean ± standard deviation (SD), and comparisons between two and three independent groups were performed using independent *t* test and one-way ANOVA with Tukey’s post-hoc for multiple comparisons, respectively. The relative expression of miR-452-3p and STAT3 mRNA did not fit a Gaussian distribution and was presented as median (1st quartile–3rd quartile), with the comparisons between two and three groups being performed by Mann–Whitney U-test and Kruskal–Wallis test, respectively. Dunn’s test was performed for pair-wise comparisons. For categorical variables, frequencies were analyzed by the chi-square (χ^2^), with odds ratios (OR) and the 95% confidence interval (CI) being calculated. In addition, compatibility of genotype distribution with Hardy–Weinberg equilibrium was tested using the chi-square test. When analyzing correlations, Spearman rank correlation (rs) was used for non-parametric variables and Pearson correlation (r) for parametric variables. A probability value (*p* < 0.05) was statistically significant.

## 3. Results

### 3.1. Demographic and Biochemical Characteristics of the Studied Groups

Both groups (patient and control groups) were age (*p* = 0.746)-, sex (*p* = 0.787)-, and body mass index (BMI) (*p* = 0.843)-matched (Table 4). 

**Figure 2:**
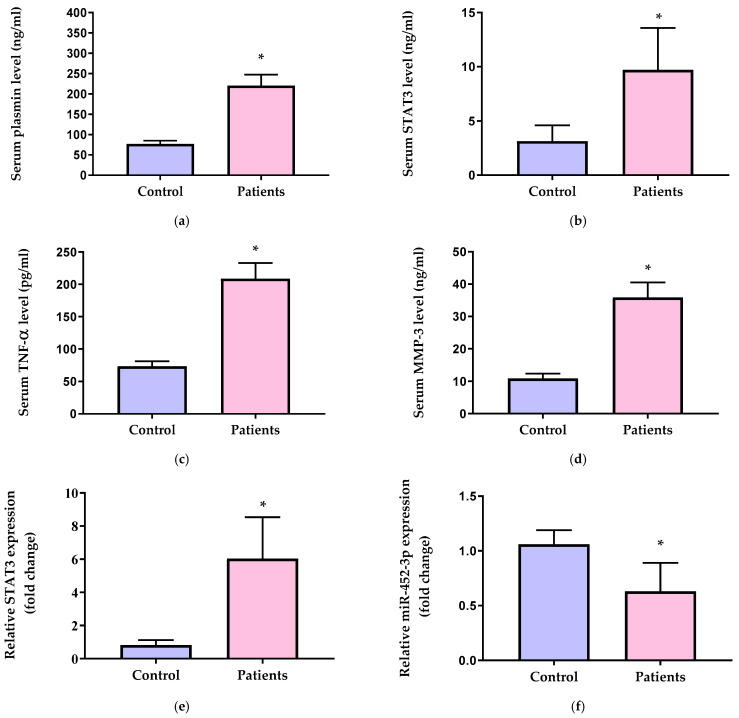
Serum levels of (**a**) plasmin, (**b**) STAT3, (**c**) TNF-α, and (**d**) MMP-3 and relative gene expression of (**e**) STAT3 and (**f**) miR-452-3p in the control group and OA patients. STAT3 = signal transducer and activator of transcription 3, TNF-α = tumor necrosis factor alpha, and MMP-3 = matrix metalloproteinase-3. Data of serum levels of biochemical parameters are presented as mean ± SD and compared by unpaired *t* test. Expression values of STAT3 and miR-452-3p are presented as median (IQR) and compared by Mann–Whitney test. * Significantly different vs. control at *p* < 0.05.

Serum plasmin (Figure 2a), STAT3 (Figure 2b), TNF-α (Figure 2c), and MMP-3 levels (Figure 2d) were significantly elevated in OA patients compared to healthy control individuals (*p* < 0.001). OA patients exhibited upregulated STAT3 mRNA expression (6.04-fold) (Figure 2e) and downregulated miR-452-3p expression (0.63-fold) (Figure 2f) compared to the control group (*p* < 0.001).

### 3.2. The Association of STAT3 and miR-452-3p Expression Levels with the Clinical Scores and Biochemical Markers in OA Patients

As shown in Table 5, the gene expression of STAT3 in the serum of OA patients was positively correlated with NRS values (*p* < 0.001) and negatively correlated with all the KOOS subscales (*p* < 0.001), except for the subscale of symptoms other than pain (*p* = 0.117). The serum protein levels of STAT3 were also negatively correlated with all KOOS subscales. Moreover, STAT3 gene expression and protein levels showed positive correlation with each other (*p* < 0.001), and each of them was positively correlated with the concentrations of the inflammatory markers including plasmin, TNF-α, and MMP-3 (*p* < 0.001).

Conversely, the expression of miR-452-3p showed significant negative correlation with NRS score (*p* < 0.001), but it was positively correlated with the KOOS subscales (*p* < 0.001), except for the symptoms other than pain subscale (*p* = 0.089). The expression levels of miR-452-3p were negatively correlated with the serum concentrations of STAT3, plasmin, TNF-α, and MMP-3 (*p* < 0.001) (Table 5).

### 3.3. Molecular Analysis of rs1053005 Variant of STAT3 Gene in OA Patients

The genotype distribution of STAT3 rs1053005 polymorphism among patients and controls matched predictions made by the Hardy–Weinberg equilibrium (*p* = 0.264). Compared with the major AA genotype, the rare GG genotype was more frequent in the control group than in the OA group (15.5% vs. 5.8%) [OR (95% CI) = 0.31 (0.16–0.60); *p* < 0.001]. In accordance, the major high-risk A allele of the STAT3 gene rs1053005 (A/G) SNP was more prevalent in the OA patients than in the control group (74.4% vs. 64.5%), whereas the minor low-risk G allele was more frequent in the control group than in the OA patients (35.5% vs. 25.6%), suggesting a protective effect against OA incidence [OR (95% CI) = 0.62 (0.47–0.83); *p* < 0.001] (Table 6).

### 3.4. STAT3 rs1053005 Polymorphism Association with Serum Levels of Plasmin, STAT3, TNF-α, MMP-3, and STAT3, miR-452-3p Expression in OA Patients

**Figure 3:**
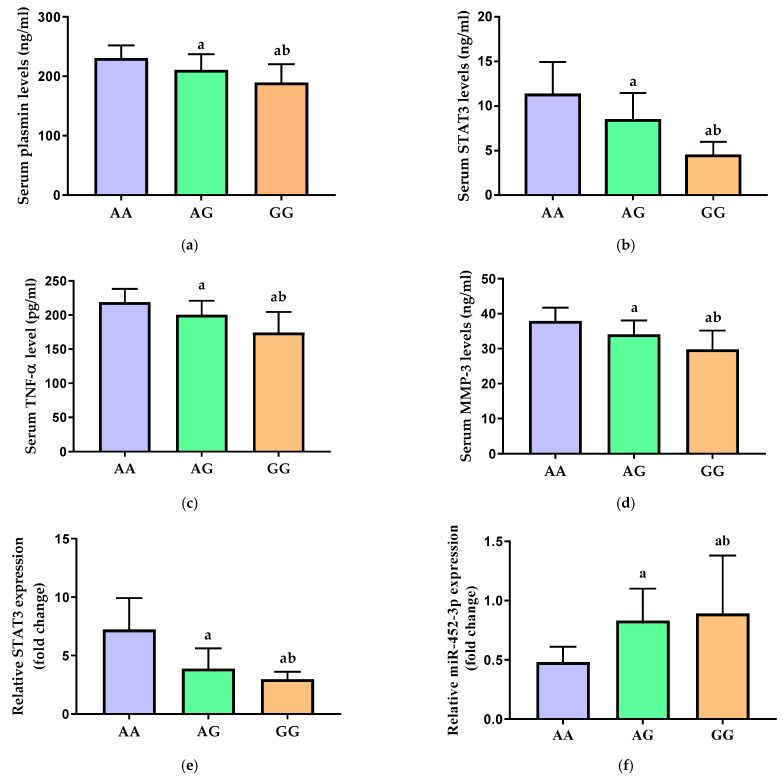
Serum levels of (**a**) plasmin, (**b**) STAT3, (**c**) TNF-α, and (**d**) MMP-3 and relative gene expression of (**e**) STAT3 and (**f**) miR-452-3p in carriers of the different genotypes of STAT3 rs1053005 polymorphism. STAT3 = signal transducer and activator of transcription 3, TNF-α = tumor necrosis factor alpha, and MMP-3 = matrix metalloproteinase-3. Data of serum levels of biochemical parameters are presented as mean ± SD and compared by one-way ANOVA. Expression values of STAT3 and miR-452-3p are presented as median (IQR) and compared by Kruskal–Wallis test. a Significantly different vs. AA carriers at *p* < 0.05; b significantly different vs. AG carriers at *p* < 0.05.

In comparison to the high-risk AA genotype, the carriers of the minor G allele in both the heterozygote (AG) and the homozygote (GG) genotypes had significantly lower serum concentrations of plasmin (Figure 3a), STAT3 (Figure 3b), TNF-α (Figure 3c), and MMP-3 (Figure 3d) (*p* < 0.001). Their levels in the carriers of the homozygote GG genotype were significantly lower than those in the heterozygote AG carriers (*p* < 0.001). 

Additionally, the relative expression of STAT3 mRNA in the serum was significantly decreased in the AG and GG carriers as compared to the major AA genotype carriers (*p* < 0.001) (Figure 3e). There was a significant increase in the relative expression of miR-452-3p in both AG and GG genotypes carriers when compared with the high-risk AA genotype of STAT3 rs1053005 polymorphism in OA patients (*p* < 0.001) (Figure 3f). Interestingly, the lowest STAT3 gene expression and the highest miR-452-3p expression were observed in patients with the recessive GG genotype.

### 3.5. Assessment of the Therapeutic Efficacy of HMW-HA Intra-articular Injection in OA Patients

To assess the therapeutic efficacy of HMW-HA intra-articular injection in OA patients, the mean clinical NRS and KOOS scores were compared between patients who received HMW-HA intra-articular injection (*n* = 83) and those who did not receive it (*n* = 175). The serum levels of STAT3 protein, STAT3 mRNA, and miR-452-3p as well as the mean NRS and KOOS scores were compared both at the baseline (i.e., before injection) and after 6 months following the HMW-HA injection (Table 7). At baseline (before injection), none of the assessed parameters or scores showed a significant difference between both groups. However, at the ultimate follow-up (i.e., six months after the injection), patients who received the HMW-HA injection showed significantly lower STAT3 concentrations (*p* = 0.040), lower STAT3 mRNA expression (*p* = 0.014), and higher miR-452-3p expression (*p* = 0.020) compared with patients who did not receive HMW-HA injection. Additionally, a significantly lower mean NRS score and significantly higher mean KOOS subscales were recorded in patients who received HMW-HA injection relative to the patients who did not receive it (*p* < 0.01) (Table 7), indicating a significant improvement. 

For further assessment of the efficacy of HMW-HA injection in OA patients, the percentage change in NRS and KOOS clinical scores was calculated in each patient using the equation: % change score = (score after 6 months − score at baseline)/score at baseline) × 100. The % change in STAT3 concentration, STAT3 gene expression levels, and miR-452-3p expression after HMW-HA injection was calculated similarly. The median (1st–3rd quartiles) of the percentage change in each of the two groups was calculated and statistically compared. The serum levels of STAT3 protein and mRNA significantly decreased in the group that received HMW-HA injection by 16.99% and 35.85%, respectively, compared with a decrease by 8.43% and 25.50%, respectively, in patients who were not injected with HMW-HA (*p* < 0.01). Additionally, the NRS score significantly decreased in patients who received HMW-HA injection by 28.57%, whereas the percentage increases in all the KOOS subscales in this group were significantly higher than those in the group that did not receive the injection (*p* < 0.01) (Table 7).

### 3.6. Assessment of the Therapeutic Response to HMW-HA Intra-Articular Injection in Different Geno-Types of STAT3 rs1053005 Polymorphism in OA Patients

To assess the effect of STAT3 rs1053005 on the efficacy of HMW-HA intra-articular injection in OA patients, the percentage changes in serum levels of STAT3 protein, STAT3 mRNA expression, and miR-452-3p expression, as well as the percentage change in NRS and KOOS scores (before and six months after injection), were compared between the carriers of the high-risk major AA genotype and the carriers of both AG and GG genotypes. The median percentage decrease in STAT3 concentrations and STAT3 gene expression was significantly more pronounced in carriers of AG and GG genotypes than in carriers of the AA genotype (*p* < 0.001). However, the percentage increase in miR-452-3p expression was significantly higher in the carriers of the G allele (i.e., AG and GG genotypes) relative to AA genotype (*p* < 0.001) (Table 8).

Moreover, the NRS score decreased in the carriers of the AA genotype by 16.67%, but the median percentage decrease in carriers of the low-risk G allele in both the heterozygote and the homozygote (AG + GG) genotypes was 40% (*p* < 0.001). In accordance, the percentage increase in all the subscales of the KOOS score in AG + GG genotype carriers was significantly higher than that in the carriers of AA genotype (*p* < 0.001), indicating a more efficient improvement in carriers of STAT3 rs1053005 AG + GG genotypes vs. carriers of the dominant AA genotype (Table 8). 

## 4. Discussion

Osteoarthritis is the most prevalent type of arthritis, accounting for most of the pain, disability, and impaired adult work performance globally [27]. The diagnosis of OA is still mostly based on plain radiography. The KL grading system uses radiographic images from X-rays for classification of knee OA severity into 5 grades, from grade 0 (normal) to grade 4 (severe), based on the extent of joint space narrowing and the presence of osteophytes [23]. KL-graded scores were either 1, 2, or 3 in all patients included in this study. In addition, functional assessment of OA symptoms using NRS and KOOS scoring systems was performed. The mean NRS of OA patients in this study was 5.58 ± 0.69, indicating moderate to high pain levels, and the mean KOOS was 58.83 ± 8.37 for pain, 68.15 ± 8.26 for other symptoms, 68.41 ± 7.76 for function in daily life, 30.30 ± 4.27 for function in sports and recreation, and 35.21 ± 4.62 for knee-related quality of life.

Given that age, sex, and BMI are considered as strong risk factors for OA [28,29,30], participants in the current study in both patient groups and control groups were age-, sex-, and BMI-matched to exclude the interference of such factors with current study results.

Osteoarthritis affects the synovial joints, with failure of the articular cartilage and thickening of the subchondral bone. Such events can be driven by a complex interaction between genetic, metabolic, biochemical, and biological variables, along with inflammatory components [31]. Large amounts of inflammatory cytokines (IL-1, IL-6, TNF- α, and IL-8) are produced by the synovial membrane and diffused into the cartilage through the synovial fluid, activating the chondrocytes to produce additional proinflammatory cytokines that speed up cartilage degradation and OA progression [32]. The effect of these cytokines is transmitted through several signaling pathways. Among these pathways, the STAT3 signaling pathway plays a crucial role in OA development and progression via the induction of cytokine production and immune responses [33]. 

It has been demonstrated that chondrocytes from OA patients exhibit higher STAT3 expression than chondrocytes from healthy controls [34]. It has also been found that OA cartilage tissue had considerably higher STAT3 expression levels than normal cartilage tissues in a mouse model of collagenase-induced OA. Those elevated STAT3 expression levels have been reported to be associated with reduced collagen type-2 levels and damaged cartilage matrix [35,36]. Consistent with these findings, our results showed a significant increase in serum mRNA and protein levels of STAT3 in OA patients compared to healthy individuals, indicating deregulated STAT3 activation.

It is worth mentioning that enhanced STAT3 activation collectively intensifies the inflammatory symptoms of OA and decreases joint performance through increased production of proinflammatory cytokines such as TNF-α, upregulated expression of degradative MMPs, and enhanced degradation of cartilage components [37]. Previous studies showed that TNF-α expression levels were increased in cartilage cells from patients with OA [38,39]. TNF-α upregulates the production of inflammatory cytokines and enzymes such as IL-6, IL-8, VEGF, iNOS, COX-2, and PGE-2 synthase. Moreover, TNF-α prevents the synthesis of type II collagen and proteoglycan components by chondrocytes and promotes the breakdown of the ECM by activating aggrecanases and collagenases such as MMP-1, MMP-3, and MMP-13 [40]. 

Elevated MMP-3 levels damage endothelial cell connections and break down collagen in the ECM, with the inflammatory cells being more prone to permeate. Therefore, OA progresses in a vicious cycle in which MMP-3 increases synovitis, and synovitis enhances MMP-3 production. Indeed, proliferating synovial tissues have been reported to exhibit elevated levels of MMP-3 expression [41].

Another inflammatory mediator that contributes to OA pathogenesis is the plasminogen/plasmin system. Plasminogen is activated to yield the serine protease plasmin, a key enzyme that has been shown to mediate ECM remodeling either directly or indirectly by cleaving various ECM proteins and activating latent MMPs [42,43]. Studies have shown that OA cartilage exhibits approximately five times higher plasmin activity than controls [42,44]. Consistent with the previous findings, our results showed a significant increase in TNF-α, MMP-3, and plasmin serum levels in OA patients compared to the healthy control group. 

Moreover, the current results revealed that STAT3 mRNA and protein levels were positively correlated with NRS values and negatively correlated with all the KOOS subscales, except for the subscale of symptoms other than pain. There has also been a positive correlation between STAT3 mRNA and protein levels and the concentrations of the inflammatory markers including plasmin, TNF-α, and MMP-3, with STAT3 gene expression being also positively correlated with its serum concentrations.

Previous studies have suggested the involvement of miRNA in the regulation of the balance between the development, homeostasis, and destruction of articular cartilage that occurs through regulating OA-related genes by binding to the 3′-UTR of a target mRNA, with consequent translational repression and/or mRNA cleavage [15,45]. Altered expression patterns of miR-452-3p, found on human chromosome Xq28, have been observed in various disorders [10]. miR-452-3p has been previously reported to promote osteoblast differentiation, which is noted to be dysregulated in OA, exerting a key role in its pathogenesis [46]. Furthermore, downregulated miR-452 leads to an increased proinflammatory state through activation of the STAT3 inflammatory pathway [11]. Also, Lamichhane et al. [11] and Christopoulou et al. [47] have demonstrated the role of miR-452-3p in the inflammatory process and the expression of matrix metalloproteinases, the two major processes mediating OA pathogenesis. This agrees with the results of the current study, in which miR-452-3p relative expression was significantly decreased in OA patients compared to the healthy control group. Moreover, miR-452-3p expression showed a significant negative correlation with NRS scores and a positive correlation with the KOOS subscales, except for the symptoms other than pain subscale. The expression levels of miR-452-3p were negatively correlated with the serum concentrations of STAT3, plasmin, TNF-α, and MMP-3, suggesting the role of miR-452-3p in mitigating the disease progression through controlling the inflammatory process that is considered the hallmark of OA.

Based on the above results, and considering that STAT3 is a critical transcriptional factor that regulates the expression of many inflammatory mediators involved in OA pathogenesis, with miRNAs exerting a crucial role in the regulation of its expression, it is thought that SNPs in miRNA-binding sites of the STAT3 gene can significantly influence its expression and the risk of OA development. Among these SNPs, rs1053005 A/G SNP in the 3′ untranslated region of STAT3 mRNA was identified as a Hyper-IgE recurrent infection syndrome 1 susceptibility locus, which is responsible for many autoimmune disorders such as Grave’s disease and Hashimoto thyroiditis [48,49,50]. It has also been shown to be associated with type 2 diabetes mellitus [13], epilepsy [14], chronic hepatitis B virus infection [51], noise-induced hearing loss in the Chinese population [12], and systemic lupus erythematosus in the Iranian population [15]. 

To the best of our knowledge, this study is the first to assess the association between STAT3 gene polymorphism rs1053005 and the susceptibility to OA development. Moreover, this study aimed to examine the effect of rs1053005 SNP on STAT3 mRNA and protein levels, with attention being paid to the possible role of miR-452-3p.

This study revealed that the rare GG genotype and the minor low-risk G allele of the STAT3 gene rs1053005 (A/G) SNP were more frequent in the control group than in the OA group, suggesting a protective effect against OA. In accordance, there was a significant decrease in relative expression of STAT3 mRNA and STAT3 protein serum levels in the low-risk GG genotype carriers when compared with the high-risk AA and AG genotypes of STAT3 rs1053005 polymorphism in OA patients, indicating the potential impact of STAT3 rs1053005 SNP on STAT3 gene expression. Furthermore, our results showed a significant decrease in serum levels of plasmin, TNF-α, and MMP3 in low-risk GG genotype carriers compared to high-risk AA and AG genotype carriers. Based on the current results, the G allele of STAT3 rs1053005 SNP has been associated with decreased OA genetic susceptibility and severity in our study population via decreasing STAT3 gene expression and the subsequent inflammatory response.

The presence of SNPs in the 3′-UTR of mRNA can influence the regulatory activity of miRNAs on target gene expression via enhancing, disrupting, or eliminating the binding efficacy of miRNA at this region [9]. Importantly, our results showed a significant increase in the relative expression of miR-452-3p, parallel to the observed decrease in STAT3 expression, in the low-risk GG genotype carriers when compared with the high-risk AG and AA genotypes of STAT3 rs1053005 polymorphism in OA patients. Thus, it is suggested that the presence of G allele rs1053005 SNP might enhance the binding efficacy of miR-452-3p at this locus on STAT3 mRNA, leading to its degradation and decreased STAT3 levels. Based on these findings, miR-452-3p may be involved in the etiology and progression of OA and could be regarded as a promising therapeutic target in OA treatment.

Given that NSAIDs, the first medication prescribed to patients with symptomatic knee OA, are associated with an increased risk of serious gastrointestinal and cardiovascular side effects, their use should be restricted, because their safety profile conflicts with the chronic nature of OA, for which prolonged symptomatic treatment is frequently necessary [22]. Patients with knee OA were offered an alternate therapy using intra-articular injections of HA, which has similar effects as NSAID in terms of reducing pain and improving function without the aforementioned side effects [52]. Numerous systematic reviews and meta-analyses on the efficiency of intra-articular HA have been carried out following significant randomized controlled trials [53,54]. A major aim of this study was to assess the therapeutic efficacy of HMW-HA intra-articular injection in OA patients.

Eighty-three OA patients received intra-articular HMW-HA injections with outpatient visits at 2, 3, and 6 (ultimate follow-up) months, which were scheduled for follow-up of all patients. There was a significant difference between the patients who received the HMW-HA injection and the patients who did not receive it, where the HMW-HA group displayed significantly decreased STAT3 protein and mRNA levels by 16.99% and 35.85%, respectively, compared with a decrease of 8.43% and 25.50%, respectively, in the No HMW-HA group. Additionally, miR-452-3p expression was increased in the group that received the HMW-HA injection by 38.1% compared with a 20.63% increase in the group that did not receive the injection. Moreover, the NRS score significantly decreased in patients who received the HMW-HA injection by 28.57%, and the percentage increases in all the KOOS subscales in the group that received the HMW-injection were significantly higher than those in the group that did not receive the injection after a follow-up period.

To assess the efficacy of the HMW-HA intra-articular injection in different STAT3 rs1053005 genotypes in OA patients, the percentage changes in the clinical scores, STAT3 protein, and mRNA levels and miR-452-3p expression (before and six months after injection) were compared between the high-risk major AA genotype carriers and the AG + GG genotype carriers. The percentage decrease in STAT3 protein and mRNA levels, as well as the percentage increase in miR-452-3p expression, were significantly more pronounced in AG + GG genotypes carriers than in carriers of the AA genotype.

Interestingly, there was a better HMW-HA injection treatment response in OA patients carrying the low-risk G allele, as evidenced by the 40% decrease in the NRS score in carriers of the low-risk G allele in both the heterozygote and the homozygote (AG + GG) genotypes, as compared to only a 16.67% decrease in the same score in the carriers of the AA genotype. Moreover, the percentage increase in all the subscales of the KOOS score in AG + GG genotype carriers was significantly higher than that in the carriers of the AA genotype. Such improvement in the clinical scores of HMW-HA-injected patients carrying the low-risk G allele may be attributed to decrements in STAT3 protein and mRNA levels, parallel to upregulated miR-452-3p expression.

This study had a relatively small sample size and was only targeted to the Egyptian population in the Suez Canal region. Future studies that include larger sample sizes from various populations will be necessary to both confirm our findings and provide more insights on the implications of miR-452-3p in OA pathogenesis and the precise mechanism underlying its impact on STAT3 expression. Additionally, more similar studies can help to confirm the association of the STAT3 rs1053005 genetic variant and other STAT3 genetic variants with OA susceptibility and severity. The buildup of additional data from future studies will support more comprehensive understanding of the mentioned associations and enable the development of novel therapeutic strategies.

## 5. Conclusions

The current study demonstrated that decreased miR-452-3p and increased STAT3 expression levels were associated with increased proinflammatory cytokines, upregulated expression of degradative MMPs, and degradation of cartilage components, which collectively intensified OA inflammatory symptoms and decreased joint performance. Also, STAT3 rs1053005 SNP was associated with decreased OA genetic susceptibility in the current study population as a result of increased miR-452-3p and decreased STAT3 expression. In addition, the current results revealed improvement in OA patients’ clinical scores six months after HMW-HA injection, with a more efficient improvement being observed in carriers of the STAT3 rs1053005 AG + GG genotypes vs. carriers of the dominant AA genotype, which may be attributed to the more apparent decrease in STAT3 protein and mRNA levels, and the increase in miR-452-3p expression in HMW-HA-injected patients carrying the low-risk G allele may be attributed to decrements in STAT3 protein and mRNA levels, parallel to upregulated miR-452-3p expression.

## Figures and Tables

**Table 1:** Kellgren and Lawrence osteoarthritis grading system criteria.

Grade 0	No joint space narrowing (JSN) ^1^ or reactive changes
Grade 1	Possible osteophytic lipping + doubtful JSN
Grade 2	Definite osteophytes + possible JSN
Grade 3	Moderate osteophytes + definite JSN + some sclerosis + possible bone end deformity
Grade 4	Large osteophytes + marked JSN + severe sclerosis + definite bone end deformity

^1^ JSN: joint space narrowing.

**Table 2:** Clinical scores of the OA patients (*n* = 258).

Clinical Scores	Mean ± SD	Min–Max
NRS (0–10)	5.58 ± 0.69	4.0–7.0
KOOS subscales (0–100)		
Pain	58.83 ± 8.37	38.0–76.0
Other symptoms	68.15 ± 8.26	50.0–84.0
Function in daily life	68.41 ± 7.76	50.0–82.0
Function in sports and recreation	30.30 ± 4.27	22.0–40.0
Knee-related quality of life	35.21 ± 4.62	25.0–46.0

NRS = the Numeric Rating Scale, KOOS = Knee injury and Osteoarthritis Outcome Score. Data are presented as mean ± SD.

**Table 3:** Sequence of the primers used for real-time PCR.

Gene	Primer Sequence	Annealing Temp.
STAT3	Forward primer: 5′-CAGCAGCTTGACACACGGTA-3′ Reverse primer: 5′-AAACACCAAAGTGGCATGTGA-3′	52 °C
GAPDH	Forward primer: 5′-GGAGCGAGATCCCTCCAAAAT 3′ Reverse primer: 5′-GGCTGTTGTCATACTTCTCATGG-3′	56 °C
miR-452-3p	Forward primer: 5′-GCGAACTGTTTGCAGAGG-3’ Reverse primer: 5′-CAGTGCGTGTCGTGGAGT-3’	50 °C
U6	Forward primer: 5’-CTCGCTTCGGCAGCACA-3’ Reverse primer: 5’-AACGCTTCACGAATTTGCGT-3’	50 °C

**Table 4:** Demographic and biochemical characteristics of the control group and OA patients.

Variables	Control (*n* = 200)	OA Patients (*n* = 258)	*p* Value
Number (%)			
Sex			
Male	99 (49.5%)	131 (50.8%)	0.787
Female	101 (50.5%)	127 (49.2%)	
Mean ± SD			
Age (years)	46.93 ± 11.88	47.26 ± 9.19	0.746
BMI (kg/m^2^)	28.4 ± 3.93	28.47 ± 3.87	0.843

Data of sex distribution were compared by chi-square test. Data of age and BMI were compared by unpaired *t* test. BMI = body mass index; SD, standard deviation.

**Table 5:** Correlation of STAT3 gene expression levels, STAT3 protein level, and miR-452-3p expression levels with the clinical scores and biochemical parameters in the OA patients (*n* = 258).

	STAT3 Serum Protein Levels		STAT3 Expression		miR-452-3p Expression	
	R	*p*-Value	r_s_	*p*-Value	r_s_	*p*-Value
Clinical Scores						
NRS (0–10)	0.116	0.063	0.320	0.001 *	−0.205	0.001 *
KOOS subscales (0–100)						
Pain	−0.201	0.001 *	−0.383	0.001 *	0.245	0.001 *
Other symptoms	−0.178	0.004 *	−0.098	0.117	0.106	0.089
Function in daily life	−0.190	0.002 *	−0.380	0.001 *	0.243	0.001 *
Function in sports and recreation	−0.216	0.001 *	−0.400	0.001 *	0.258	0.001 *
Biochemical markers						
STAT3 (ng/mL)	--	--	0.586	0.001 *	−0.581	0.001 *
Plasmin (ng/mL)	0.838	0.001 *	0.523	0.001 *	−0.611	0.001 *
TNF-α (pg/mL)	0.856	0.001 *	0.532	0.001 *	−0.610	0.001 *
MMP-3 (ng/mL)	0.766	0.001 *	0.523	0.001 *	−0.660	0.001 *

NRS, the Numeric Rating Scale; KOOS, Knee injury and Osteoarthritis Outcome Score; STAT3, signal transducer and activator of transcription 3; TNF-α, tumor necrosis factor alpha; MMP-3, matrix metalloproteinase-3. r, Pearson’s correlation coefficient; r_s_, Spearman’s correlation coefficient. * Significantly correlated at *p* < 0.05.

**Table 6:** Allele frequencies and genotype distribution of STAT3 rs1053005 (A/G) polymorphism in the control group and in OA patients.

	Control (*n* = 200)	Patients (*n* = 258)	*p* Value	OR (95%CI)
STAT3 rs1053005				
Genotypes				
AA	89 (44.5%)	141 (54.7%)		
AG	80 (40%)	102 (39.5%)	0.281	0.80 (0.54–1.19)
GG	31 (15.5%)	15 (5.8%)	0.001 *	0.31 (0.16–0.60)
Alleles				
A	258 (64.5%)	384 (74.4%)		
G	142 (35.5%)	132 (25.6%)	0.001 *	0.62 (0.47–0.83)

Data are represented by number (%) and compared by chi-square test. STAT3, signal transducer and activator of transcription 3; CI, confidence interval; OR odds, ratio. * Indicates significant difference at *p* < 0.05.

**Table 7:** Comparison of STAT3 protein levels, STAT3 gene expression, miR-452-3p expression, and the clinical scores in patients who received HMW-HA vs. patients who did not receive HMW-HA injections (at baseline and after 6 months).

	Baseline (before Injection)		Follow-Up (after 6 Months)		% Change	
	HMW-HA (*n* = 83)	No HMW-HA (*n* = 175)	HMW-HA (*n* = 83)	No HMW-HA (*n* = 175)	HMW-HA (*n* = 83)	No HMW-HA (*n* = 175)
STAT3 (ng/mL)	9.58 ± 3.84	9.79 ± 3.88	7.93 ± 4.01	9.01 ± 3.88 *	−16.99 (−11.79–−27.18)	−8.43 (−6.85–−11.18) *
STAT3 expression (fold change)	5.44 (3.62–8.34)	6.04 (3.92–8.60)	3.49 (1.58–6.35)	4.50 (2.38–7.06) *	−35.85 (−22.76–−56.35)	−25.50 (−17.91–−39.29) *
miR-452-3p expression (fold change)	0.63 (0.43–0.96)	0.63 (0.46–0.87)	0.85 (0.60–1.32)	0.76 (0.59–1.00) *	38.10 (28.57–45.71)	20.63 (15.03–28.57) *
Clinical Scores						
NRS (0-10)	5.66 ± 0.59	5.54 ± 0.72	3.98 ± 0.98	5.24 ± 0.84 *	−28.57 (−16.67–−40.00)	0 (0–−16.67) *
KOOS subscales (0–100)						
Pain	58.04 ± 7.91	59.21 ± 8.58	63.66 ± 9.26	60.28 ± 8.77 *	8.77 (4.44–13.33)	1.85 (0–3.28) *
Other symptoms	67.43 ± 8.05	68.49 ± 8.36	73.05 ± 9.52	69.46 ± 8.52 *	8.62 (4.05–11.59)	1.54 (0–2.78) *
Function in daily life	67.17 ± 7.88	68.99 ± 7.66	72.69 ± 9.23	69.63 ± 8.07 *	7.35 (3.51–12.00)	1.33 (0–2.78) *
Function in sports and recreation	30.20 ± 4.18	30.31 ± 4.33	36.06 ± 6.29	31.11 ± 4.58 *	18.75 (8.33–26.47)	3.03 (0–6.25) *
Knee-related quality of life	35.92 ± 4.58	34.87 ± 4.61	41.72 ± 7.16	35.76 ± 4.93 *	15.15 (7.41–22.50)	2.94 (0–5.26) *

HMW-HA, high-molecular-weight hyaluronic acid; NRS, the Numeric Rating Scale; KOOS, Knee injury and Osteoarthritis Outcome Score; % change score, (score after 6 months − score at baseline)/score at baseline) × 100; the negative sign, decreased value. Parametric data are represented as mean ± SD and compared with unpaired *t* test, and nonparametric data (i.e., data of expression levels and data of % change) are represented as median (IQR) and compared with Mann–Whitney test. * Indicates significant difference at *p* < 0.05.

**Table 8:** The % change in STAT3 protein levels, STAT3 gene expression, miR-452-3p expression, and the clinical scores in patients who underwent HMW-HA (*n* = 83) carrying different STAT3 rs1053005 genotypes.

Clinical Scores	AA (*n* = 45)	AG + GG (*n* = 38)	*p* Value
STAT3 (ng/mL)	−13.52 (−9.18–−16.63)	−27.18 (−22.07–−32.72)	0.001 *
STAT3 expression (fold change)	−23.78 (−19.74–−28.89)	−59.13 (−51.59–−68.46)	0.001 *
miR-452-3p expression (fold change)	34.78 (28.57–40.00)	45.42 (25.68–59.00)	0.001 *
NRS (0–10)	−16.67 (−16.67–−20.00)	−40.00 (−33.33–−50.00)	0.001 *
KOOS subscales (0–100)			
Pain	4.62 (3.13–8.80)	13.05 (9.94–16.67)	0.001 *
Other symptoms	4.55 (3.80–8.62)	11.57 (8.93–13.76)	0.001 *
Function in daily life	3.80 (2.63–7.48)	11.30 (8.66–14.47)	0.001 *
Function in sports and recreation	8.82 (6.70–15.10)	25.00 (22.05–33.57)	0.001 *
Knee-related quality of life	7.50 (5.36–12.31)	23.02 (17.84–29.02)	0.001 *

Data are presented as median (1st–3rd quartiles) and compared with Mann–Whitney test. NRS, the Numeric Rating Scale; KOOS, Knee injury and Osteoarthritis Outcome Score. * Indicates significant difference at *p* < 0.05.

## Data Availability

Data generated and analyzed during this study are included in the published article.
